# Multiple group membership, social network size, allostatic load and well-being: A mediation analysis

**DOI:** 10.1016/j.jpsychores.2021.110636

**Published:** 2021-12

**Authors:** Gallagher Stephen, T. Muldoon Orla, M. Bennett Kate

**Affiliations:** a Department of Psychology, Centre for Social Issues Research, Study of Anxiety, Stress and Health Laboratory, University of Limerick, Limerick, Ireland; b Health Research Institute, University of Limerick, Castletroy, Limerick, Ireland; c Department of Psychology, University of Liverpool, Eleanor Rathbone Building, Bedford Street South, Liverpool, UK.

**Keywords:** Allostatic load, Group membership, Social cure, Social relationships, Well-being

## Abstract

**Objectives:**

This study examined whether social network size and allostatic load (AL) mediated the association between multiple group membership (MGM) and future physical and psychological well-being.

**Methods:**

A longitudinal design was used and data from 1026 healthy participants on the relevant variables was extracted from Wave 2(2010−12), Wave 3 (2011−2013) (for baseline MGM, social network size and AL) and Wave 9 (2017–19) (for well-being at follow-up) of the Understanding Society UK population-based dataset.

**Results:**

MGM was not directly associated with future well-being, but both social network size, *β* = 0.06, *t* = 2.02, *p* = .04, and AL, *β* = −0.06, *t* = −2.05, *p* = .04, were associated with physical but not psychological well-being at follow-up. Those who had higher numbers of friends had better physical well-being, and those who had lower AL risk scores had better physical well-being at follow-up. However, MGM was indirectly associated with physical well-being through social network size, and AL such that those reporting higher MGM, reported a greater number of friends which was associated with a lower AL and then future physical well-being, β = 0.004, CI [0.001., 0.0129]. This was not evident for psychological well-being. This mediation withstood adjustment for confounding factors (e.g. age, gender, marital status lifestyle factors).

**Conclusion:**

The present study extends findings on the existing social relationships and social cure literature and our findings are discussed in relation to the social cure hypothesis.

## Highlights

• Multiple group membership is indirectly associated with better future well-being.
• Pathways behind this association are high social network size and lower allostatic load.
• High group membership increased network size which was linked to lower allostatic load.

## 1 Introduction

The importance of social factors for health and well-being is now well-established [22,45,54]. For example, people who are less socially connected relative to those who are more connected are at a higher risk of poorer physical and psychological well-being including lower quality of life [55]. Allostatic load, which is conceptualised as a measure of the cumulative burden on multiple physiological systems (e.g. metabolic, immune and endocrine) on the body as it attempts to adapt to life's demands [37] is one biological pathway behind the support relationships and well-being associations [41,54,62]. For example, a recent review of the literature has found that greater social relationships were associated with reduced allostatic load (AL) [60]; and as reported by Guidi and colleagues, higher purpose in life, a dimension of well-being, predicted lower levels of AL [15]. As such, it is likely that social relationships affects well-being via AL. This is the focus of the present study. It is also worth noting that both physical and psychological well-being are associated cardiovascular disease morbidity and mortality [43,64] and all-cause mortality [40].

The theory of ‘allostatic load’ (AL) has been proposed as a model of understanding the physical and mental health consequences of dealing with the prolonged stress [35]. This theory posits that repeated or inadequate physiological adaption to stress over time results in *wear and tear* on the body and brain, which mediated through the dysregulation of glucocorticoids via the hypothalamic-pituitary-adrenal (HPA) axis and catecholamine via the sympathetic nervous system (SNS), will result in dysfunction of the cardiovascular, immune, and metabolic systems [35,36], thereby leading to ill-health. AL confers and can often be a precipitative factor for mental and physical health outcomes longitudinally [28]. This cumulative burden of AL, in turn, causes *wear and tear* on the body/brain and has been found to predict coronary heart disease morbidity [48], and mortality [23], all-cause mortality [42] and dimensions of physical and psychological well-being [15]. AL is frequently operationalized as a composite index of several biomarkers from several physiological systems capturing primary (e.g., dehydroepiandrosterone (DHEA), norepinephrine) and secondary mediators (e.g., insulin-like growth factor 1(IGF-1), cholesterol, c-reactive protein (CRP)) [60]. High-risk profiles are defined as the upper (or lower) quartile, i.e. above 75% or below 25%, on these indices [23]. In relation to theoretical understanding, the model of AL proposes that dysregulation occurs as we respond or adapt to life challenges [37] Both the cognitive model [66] and social identity [67] models of stress allow for attenuation of the harmful effects of stress via stronger social relationships. Other approaches to AL go beyond the cumulative biological assessment of, a clinimetric approach for example in a clinical setting would also assess allostatic overload (AO); this refers to a state due to the cumulative interactions of life events and social stressors that are influenced by social and family contexts by exceeding the individual's resources, may constitute a danger to health [9]. This notion has been supported by more recent empirical and theoretical work on AL [15]. Though ideas of this type have taken hold in medicine and sociology [68], further integration of biological and psychological theories to create a greater understanding of the processes underlying the association between social factors and health.

This buffering effect of social relationships is also thought to attenuate or diminish physiologic reactivity to stress as well as having a positive influence on AL via improved coping and stress reappraisal [32,60].

The influence of social relationships for AL has been widely researched. For example, among older adults, those with stronger social ties with close friends and/or neighbours had lower AL [46]. Further, while negative social support (e.g. critical friends) was associated with higher AL, those who had positive support, both structural (e.g. frequency of contact) and functional (e.g. emotional support) had lower AL [5]. Similar patterns have also been confirmed in two recently published systematic reviews on positive psychosocial factors including social relationships and AL [32,60]. While there are direct associations with AL, less attention has been paid to other interactive pathways behind this association. This research on social relationships and AL has tended to examine social factors at an individual level, such as perceived social support, social isolation or socioeconomic position [60], less attention has been paid to group level processes (Haslam et al., 2021). In fact, recent research has called for a more detailed examination and application of the ‘social’ element of biopsychosocial model of health [17].

One group level factor that has not received much attention in the context of AL is multiple group membership (MGM). Group membership, while it is related to the health benefits associated with structural social support, has its origins in the social cure hypothesis, [26]. As a rule of thumb, and in line with the social cure hypothesis [26], if you belong to no groups but decide to join one, you cut your risk of dying over the next year in half [69]. The social cure is derived from the social, psychological and material resources that derive from our relationships with others via group memberships [18]. In fact, recent work has highlighted that the nature and number of group memberships is important for collective self-esteem [49], self-reported measures of health [31,58], and cardiovascular responses to stress [11]. Membership of multiple groups is not only associated with better mental health, higher self-esteem, and physical resilience [27], it is also related to better quality of life [16] and reduced stress [24] and mortality [51]. Others research has found activation in the brain's ventral medial prefrontal and anterior and dorsal cingulate cortex that correspond with group memberships [39], suggestive of a biological underpinning of group membership.

In terms of AL parameters, more recent work has found that members of closely-knit groups have lower levels of fibrinogen compared to those less closely knit. [30]. Moreover, others have found that high scores on the social network index, which assesses participation in 12 types of social relationship (e.g. spouse, parents, family members, close neighbours, friends, workmates, schoolmates, fellow volunteers, members of groups without religious affiliation, and religious groups) was associated with lower interleukin-6 [33]. While in a recent population-based study found that higher levels of participation in various groups (e.g. tenant associations, political parties, clubs and societies) was associated with lower levels of C-reactive protein(CRP), [57] an inflammatory biomarker used in the cumulative index of AL. In another study, in patients living with cancer those in 4 or more groups had lower CRP [63]. Taken together, given the weight of evidence behind MGM, health and well-being and AL indices the underlying pathways are worth investigating. In fact, the mechanisms linking MGM to health and well-being are not fully understood [49].

One way by which MGM exerts its influence on AL is through increasing social support. For example, the positive effects of MGM is through embedding people more firmly in their social world, and providing them with multiple connections to similar others [18]. Studies have also found that MGM is predictive of increased social support, especially in those who have strong group ties [49]. Moreover, given that social support can also have direct effects on physiological systems [53] it is likely relevant here. There is also evidence to show that social support is a mediating factor between MGM and well-being. In a study of Western and Asian populations, the association between MGM and well-being was mediated by increased social support [7], albeit this was only evident for Western, rather than Asian, populations. More recently, the association between group membership and COVID-19 related anxiety was mitigated by increased social support from their groups [1]. It is also likely that being a member of several groups rather than one will increase the number of friends in one's social circle/social network is much larger. Thus, while MGM may provide greater availability of social support there is also likely to be more opportunities to connect and establish relationships and friendships and as such increase your personal social network [3]. And, having a greater number of friends is associated with lower morbidity and mortality [22], a better antibody response to vaccination [13]; antibody response to vaccination is one of the best models for examining the effects of psychosocial factors on immunity in vivo [6]. A recent review of the literature has also confirmed that social network size, including number of friends, is associated with lower AL [32], suggesting its importance in this context. While both MGM and social network size are likely to have direct effects on well-being and AL, it is also likely that MGM is influencing well-being indirectly via an increasing social network and AL. It is plausible that the association between MGM and well-being is serially mediated through an individual's social network size and AL. This will be assessed in the current study.

Integrating a biological, with social psychological perspective here we use a longitudinal design, with data extracted from Wave 2 (2010–12) and Wave 9 (2017–19) of the *Understanding Society* population-based study from the UK [56], to test a mediational pathway. Based on the above literature, we hypothesised, that 1) MGM, social network size, and AL at Wave 2 (baseline) will be independent predictors of physical and psychological well-being at Wave 9 (follow-up); 2) the association between baseline MGM and well-being at follow-up will be mediated by both social network size and AL. Specially, we expect that those with greater MGM, will report higher numbers of friends and have lower AL(baseline factors) will be associated with better physical and psychological well-being at follow-up.

## 2 Methods

### 2.1 Participants

We analyzed (*N* = 1050) data extracted from Wave 2 (MGM), Wave 3 (social network size, baseline physical and mental well-being), Biomarker data from Wave 2 (Nurse study), and follow-up well-being at Wave 9 of the *Understanding Society* dataset from the UK [56]. This is a population level study of over 40,000 people living in the UK, to help understand social and economic changes on well-being in the UK. Survey data for Wave 2 was collected between 2010 and 2012; and these respondents were invited to take part in a biomarker study if they were aged 16–64 years, lived in England, Wales, or Scotland, and were not pregnant. Wave 3 data was gathered between 2011 and 2013. While over 10,000 participants took part in the biomarker study, we only included those who had data on MGM, well-being measures at both time-points (Wave 3 and Wave 9 follow-up), who were healthy (not currently ill or disabled) and not any medication (except the oral contraceptive). In terms of illness/disability, this included illnesses such as cancer, heart disease, arthritis, multiple sclerosis, Parkinson, asthma and depression. These latter exclusion criteria are pertinent as they could influence AL biomarkers and are potential well-being confounders [15]. The group membership and well-being measures while assessed during the same time window were not collected on the day of blood collection, they were done separately perhaps as a way of reducing burden on the participants. The results section has further participant information including that on other sociodemographics such as age, gender, marital status (married/partnered vs single/widowed), social class (manual v non-manual), education (University/College vs High School diploma), ethnicity (white/other ethnicities), monthly income in pounds sterling, whether they had friends or not (yes/no), health behaviors such as smoking (yes/no), walking (days walking for 30 min per day per month), number of days you had alcohol in the week, and portions of fruit and vegetables per day. These socio-demographics and health behaviors are also likely to influence health and so were controlled for in our analyses as possible covariates. The relevant longitudinal sampling percentage weighting was applied [56]. After screening out those who ill or on medication, and including only those who had the well-being measures for both time-points, we were left with a final sample of 1050 participants for analysis. Each participant gave informed consent and ethical approval for the Understanding Society nurse visit was obtained from the National Research Ethics Service (Reference: 10/H0604/2) in the UK.

### 2.2 Multiple group membership (MGM)

At Wave 2, participants were asked using a yes/no format whether they were involved in any groups, and if so to indicate memberships from a list (e.g. such as parents' association, a union, political parties, several community groups, women's institutes, or sports groups). There was a list of seventeen possible groups in total which were summed to give a frequency score of MGM. In order to include the ‘not involved in any group in our MGM index, we pooled this ‘no group’ with the number of group memberships so that possible scores went from 0 to 17. This approach to capturing group membership has been used in similar studies [31,50].

### 2.3 Social network size

This was captured once and in Wave 3, participants were also asked how many close friends they had. This was opened-ended and they could give the total number. This is a similar method for assessing personal social network size [13].

### 2.4 Allostatic load

In Wave 2 (Nurse Study) a biomarker study took place 6-months after the survey data collection of Wave 2. This biomarker dataset was collected only once in the Understanding Society dataset but allowed us to generate our AL indices. AL was defined in accordance with the initial definition [47]. Here we used 12 biomarkers representing four physiological systems: the neuroendocrine system (DHEA-s); the immune system (insulin-like growth factor-1 (IGF1), C-reactive protein (CRP), and fibrinogen); the metabolic system (high-density lipoprotein (HDL), low-density lipoprotein (LDL), glycosylated haemoglobin (HbA1C), albumin, waist circumference and body mass index (BMI); and the cardiovascular system [systolic blood pressure (SBP), diastolic blood pressure (DBP)]. Each biomarker was then dichotomized into high risk versus low risk according to quartiles or sex specific risk (e.g. waist circumference) and established risk criteria (e.g. SBP/DBP 140/90 and BMI > 25). For some indices (i.e., HDL cholesterol and DHEA-S) membership in the lowest quartile corresponds to highest risk [47]. For IFG1 both high and low levels have been predictive of morbidity and mortality [38,44] thus the top and bottom quartile were classified as a risk. Membership in the upper/lower quartile is a conservative method of categorising those at high risk of disease quantitatively through their extreme levels of system activity relative to the rest of the population [48]. These were dummy coded at 1 = high risk and 0 = low risk and AL was captured by summing the number of parameters for which the subject fell into the highest risk quartile [4,19,46]. Higher scores indicate greater level of AL and the cut-off values are displayed in Table 1.

**Table 1:** Quartiles and established criteria for contributions to AL from individual biological parameters.

*Highest quartile or established criteria*
SBP (≥ 140 mmHg) / DBP (≥ 90 mmHg)
Waist circumference Male (≥0.94 cm) Women (≥0.80 cm)
Total cholesterol/HDL (≥6.3 mmol/L)
Albumin (≥47.0 g/L)
HbA1c (≥39.0 mmol/mol)
CRP (≥3.3 mg/L)
Fibrinogen (≥3.3 g/L)

*Mixed quartile and established criteria*
BMI (<18 kg & ≥25 kg)
IGF1 (≤12 & >18 nmol/L)

*Lowest quartile*
HDL cholesterol (≤1.2 mmol/L)
DHEA-S (≤1.6 umol/L)

### 2.5 Physical and psychological well-being

The 12-item short-form (SF-12) health survey [59] was used to capture physical and psychological well-being at Wave 3 and Wave 9. While devised as a measure of health-related quality of life from a physical and mental functioning standpoint, it is frequently used as a measure of physical and psychological well-being [8,52]. This scale consists of twelve items that are combined to provide two overall scores: physical and mental well-being scales. The physical scale is made up of scores that relate to physical functioning, bodily pain, general health and role limitations due to physical health [14,59]. Examples include: “In general, would you say your health is, “Excellent, Very Good, Good, Fair or Poor” and scored from 1 to 5 respectively. “During the past 4 weeks, have you had any of the following problems with your work or other regular daily activities as a result of your physical health?” “Accomplished less than you would like (yes/no)?”. For the mental scale, items captured issues relating to mental health, vitality, social functioning and role limitations due to emotional health [14]. Examples of items here were “During the past 4 weeks, how much have you felt calm and peaceful? All of the time, Most of the time, A good bit of the time, Some of the time, A little of the time, or None of the time”. These were scored on a scale from 1 to 6 respectively. Another item was “Accomplished less than you would like (yes/no)?” Physical and mental scores can range from 0 to 100; a higher score equates to a better level of physical and/or mental health [14]. The scale has excellent test-retest reliability (*r* = 0.73–0.86); as well as very good Cronbach alpha (= 0.89).

### 2.6 Analytic approach

Data were screened for assumptions of fit and had appropriate skewness and kurtosis values and revealed no outliers. Initial analyses focused on descriptives and tests of differences to examining variation on our outcomes across sociodemographics (e.g., gender, marital status) and Pearson Correlation checked for associations between covariates and our outcome variables. This was followed by two individual hierarchal linear regressions predicting physical and psychological well-being where covariates including baseline physical and psychological well-being were entered at Step1, and MGM, social network size, and AL entered simultaneously at Step 2. Given the temporal nature of our data (longitudinal design) we were able to conduct a serial mediation using model 6 of Hayes [20] PROCESS module for SPSS, with the samples bootstrapped to 5000. Temporal nature of the data is a key perquisite for mediation models [34]. In this analysis, we also controlled for the same covariates.

## 3 Results

### 3.1 Descriptive statistics

As can be seen in Table 2, the sample was middle aged, more men, predominately White and a large proportion were married and had a University level education. The majority were non-smokers and in employment. In correlation analyses, age, gender and marital status were correlated with our primary outcomes such that those older had lower physical well-being but better psychological well-being; women and those who were not married or partnered had poor psychological well-being. White people reported better physical well-being that other ethnic minorities and did those with a higher monthly income. Smokers had poorer physical and mental well-being compared to non-smokers and those eating higher fruit and vegetables had better psychological well-being.

**Table 2:** Participant socio-demographics, health behaviors correlations with study outcomes.

	Mean (SD)/%	1	2	3	4	5	6	7	8	9	10	11	12	13
1. Age	42.87(14.34)	_	0.15**	−0.41**	0.04	−0.01	−0.01	0.12*	0.26**	0.27**	−0.01	0.22**	−0.17**	0.25**
2. Gender	47.7% female		_	−0.11*	0.02	0.01	0.09*	0.24**	−0.04	0.20**	0.03	0.05	0.00	−0.15**
3. Relationship status	56.3% married			_	−0.04	−0.09*	0.01	−0.18*	−0.28**	−0.21**	0.03	0.14**	0.03	0.13**
4. Ethnicity	92.5% White				_	−0.05	0.01	0.01	−0.07	−0.02	−0.01	−0.07	0.12**	0.04
5. Education degree	43.7% Degree					_	0.05	0.01	0.01	0.11**	0.02	0.02	0.02	0.02
6. Employment	76% employed						_	−0.15**	0.06	0.03	0.02	0.07	−0.05	0.01
7. Income	£1967.34 (£1678.57)							_	0.15**	0.09**	−0.05	0.12**	0.07	0.12**
8. Smokers	18.9%								_	−0.27**	0.09	−0.01	0.11**	0.22**
9. Fruit &vegetables	3.39 (1.55)									_	0.10*	0.03	0.01	0.12**
10. Days walking (30 min)	10.97 (10.04)										_	0.04	0.02	0.01
11. Alcohol days per week	2.99(1.88)											_	0.01	−0.04
12. SF-12: Physical- follow-up	53.39 (7.27)												_	−0.25**
13. SF-12 Mental- follow-up	50.04 (9.14),													_

Correlations are *p* < .05*; *p* < .01**.

Of our predictor variables, the mean AL score was 4.35 (1.63) and this was positively correlated with age and negatively with fruit and vegetable intake. Those who were older and who ate less fruit and vegetables had higher AL. For MGM, 41% of our sample was not involved in any groups, 16% involved with at least 2 groups, with the remainder in 3 or more groups. Men and those older and married reported significantly higher MGM. In addition, those married/partnered, compared to those single/widowed/separated were also significantly more likely to have higher MGM. Moreover, those who had a University degree (education) had better physical well-being at follow-up. Those who were members of no group reported an average of 5 friends, compared to 6 friends for those who were in one of more groups.

Thus, given the correlations above these variables were treated as potential covariates and as such along with baseline well-being measures, we controlled for those significantly correlated with each outcome in our next analyses.

### 3.2 Associations between MGM, network size, AL and physical and psychological well-being at follow-up

As can be seen in Table 3 of our hierarchal regressions, entering potential covariates at Step1, and the predictors together at Step 2, the strongest predictor of physical well-being at follow-up was physical well-being at baseline. Those who reported better physical health at baseline (2010–12) also reported better physical health at follow-up (2017–19). In total, step 1 accounted for 17% of the variance (R-squared) in physical well-being at follow-up (2017–19). MGM was not associated with physical well-being. However, social network size was positively associated with physical well-being, while AL was negatively associated, i.e. greater number of friends, and those who had a lower AL score had better physical health at follow-up. These variables added an additional 1% to the variance in physical well-being at follow-up.

**Table 3:** Regression analyses predicting SF-12 Physical and Mental health in 2017–19 from sociodempgraphics, lifestyle and main predictor variables.

		β	t	*p*	ΔR^2^
SF-12 –Physical 2017–19 Step1:	Age	−0.19	−6.68	<0.001	
	Gender	0.003	0.19	0.90	
	Marital status	−0.07	−2.25	0.024	
	Incomes	−0.002	−0.57	0.047	
	Education	−0.04	1.41	0.16	
	Smoking	0.04	1.53	0.13	
	Walking	0.003	0.16	0.90	
	Fruit &vegetables	0.04	1.50	0.13	
	SF-12 physical baseline	0.25	1.84	<0.001	
Step 2:	Social network	0.06	2.04	0.038	
	AL	−0.06	−2.05	0.037	
	MGM	−0.01	−0.30	0.75	
				0.01	0.01

SF-12 –Mental 2017–19 Step1:	Age	0.17	6.14	<0.001	
	Gender	−0.10	−3.56	<0.001	
	Marital status	0.04	1.50	0.13	
	Incomes	0.04	1.37	0.16	
	Education	0.06	2.04	0.041	
	Smoking	0.02	0.64	0.51	
	Walking	0.01	0.69	0.49	
	Fruit &vegetables	−0.01	−0.39	0.69	
	SF-12 Physical baseline	0.34	11.55	<0.001	
Step 2:	Social network	−0.03	−1.27	0.20	
	AL	−0.02	−0.72	0.47	
	MGM	0.04	1.36	0.17	
				0.34	0.002

For psychological well-being at follow-up, again baseline psychological well-being proved to be the strongest predictor, i.e., those reporting better psychological well-being at baseline had better psychological well-being at follow-up. The total variance explained by Step 1 was 18% (R-squared). None of our main predictor variables were associated with psychological well-being at follow-up.

### 3.3 Mediation analysis

**Fig. 1:**
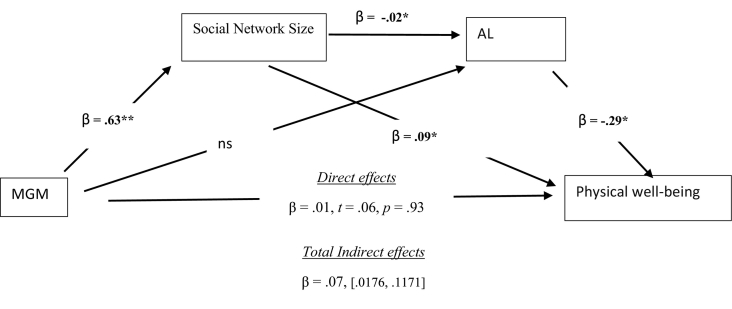
Mediation analysis of the relationship between MGM (2010–12), social network size (2011−13), AL(2011–13) and physical well-being at follow-up (2017–19). Significant effect are highlighted in bold. ** *p* < .01 level.* *p* < .05 level. Ns = non-significant; statistics refer to standardized betas (β) and 95% confidence intervals at the lower and upper limit for total indirect effects.

This serial mediation focused on physical well-being at follow-up as this was the only well-being measure associated with our predictors. In this analysis, we entered the same covariates as in the hierarchical regressions; MGM was our predictor with social network size, and AL as our mediators. A significant indirect mediation pathway was found between MGM, social network size, and physical well-being (see Fig. 1), β = 0.06, CI [0.0166,0.0974]. In addition, as predicted, we also found evidence for a serial mediation from MGM through social network size, AL and then to physical well-being at follow-up, such that those reporting higher MGM, also reported higher social networks and they had lower AL risk scores and this was associated with better physical health at follow-up in 2017–19, β = 0.004, CI [0.001., 0.0129] (see Fig. 1). The mediation from MGM to AL to physical well-being was non-significant. While our focus was on physical well-being, we re-ran the same mediation model for psychological well-being to test whether any of the pathways behind MGM and future psychological well-being were being mediated. None of the pathways were being mediated.

## 4 Discussion

The purpose of this study was to examine the association between MGM, social network size, AL and future well-being. Using a large-scale population based longitudinal study, we found that both social network and AL were associated with future physical but not psychological well-being. Although MGM was not directly associated with physical well-being at follow-up, it was indirectly associated in a serial manner, such that, those who reported being in more groups in reported more having a higher social network size i.e., they reported having more friends, and this was associated with a lower AL risk score and in turn this predicted better physical well-being at follow-up over 7 years later. This pattern was not evident for psychological well-being. It is worth noting, these were all healthy participants and free of disease at entry to the study, and given that our key findings withstood adjustment for several key socio-demographic and lifestyle factors our findings highlight the substantive health protective effects of group memberships for health.

Our findings of a positive association between AL and future physical well-being is consistent with other research showing that AL predicts poorer dimensions of physical health and well-being [15], as well other health indices [10]. Moreover, it also lends support to the notion of adopting a clinimetric approach which argeus for assessment of not just the biological aspects of AL but to combine this with the psychosocial patterns, which are more likely to be fruitful for intervention [15]. While our serial mediation model findings is in line with other social cure research [26]. Social cure research proposes that group membership is important, in that, it can facilitate increased social support, in this case a greater number of friends, which is a pathway for better well-being in general [18,25]. In this way, group memberships can be thought of as a platform for accessing social resources. The present study supports this hypothesis particularly in relation to physical well-being, but what is novel about the present study is identification of an underlying biological behind the association between MGM and well-being. In addition, our study is also consistent with other social relationship research which find support for indirect pathways underling the association with other health outcomes [21,53,60], albeit in these studies they tended to focus on personal social resources (e.g. social support and network size). Thus, our findings extend on body of work and demonstrate that MGM is a key source of social support, which can confer physiological and future health benefits. However, it must be noted that our findings were only evident for physical well-being and not for psychological well-being. One reason, although speculative on our part, for the lack of association between MGM, Social Network Size and mental well-being could be because our measurements of these constructs are not as well-validated as other studies that find such associations [2,29,31].

While our study has many strengths including a longitudinal study design, it is not without limitations. Our lack of a main effect for MGM contrasts with other social cure research on self-reported health [18] and other studies showing the benefits of MGM on biological health [57,63]. One explanation is perhaps due to methods. These previous studies not only assess group memberships but also strength of identification within those groups which is the strongest predictor of health and well-being [12,29,31]. We did not have this measure of identification, which may explain the absence of the lack of a main effect for MGM. Further, we do not know if any of our participants were cognitively impaired or had been involved in psychotherapy. Similarly, these may also underlie lack of associations with psychological well-being. Due to this dataset limitations, it is difficult to know how involved (level of meetings, types of activities) or how identified the participants were within these groups or how they experienced the group activities, something that may shed more light on our observed effects. Second, there may be other social support factors that could be important to consider such as perceived social support rather than social network size. Again, these data were not available in the Waves of interest. Third, even though our results withstood adjustment for a host of confounding factors, some of these, in particular the health and lifestyle related factors (not available in the dataset; e.g., poor sleep) could potentially be other underlying pathways [60]. These alternative pathways should be explored in future research particularly as group factors and lifestyle habits may be inextricably linked [70]. Fourth, we do not know for sure why our effects were only seen for physical well-being and not psychological well-being. It may be that attributes of social resources (e.g. quality vs quantity of groups, function vs size of network) may influence health in different ways [61], something that could be explored by future research. Finally, although the effect sizes obtained here would be considered small, it is worth noting that recent research argues that these effects are likely to be real, and help clarify and understand the actual determinants of complex psychological processes [71].

In conclusion, MGM is associated with better physical well-being in the future via an increased social network size and lower AL risk score. This pattern was not evident for psychological well-being. Our findings help to clarify and integrate the psychological and biological linkages between social factors and health, in particular of the benefits of group rather than individual level support factors [21]. Finally, our findings also speak to the importance of MGM not only for social support but also for biological health and health practitioners and policy makers can and should advise and support people to get involved with groups given these protective effects.

## Funding

This work is funded by the 10.13039/501100000781European Research Council, Advanced Grant to Professor Orla Muldoon: REF: ERC AdG 2019 SIMTIC 884927.

## Declaration of Competing Interest

We have no conflict of interest.
