# Corrigendum to “Multiple group membership, social network size, allostatic load and well-being: A mediation analysis” [Volume 151 (December 2021) 110636]

**DOI:** 10.1016/j.jpsychores.2022.111051

**Published:** 2022-12

**Authors:** Stephen Gallagher, Orla T. Muldoon, Kate M. Bennett

**Affiliations:** a Department of Psychology, Centre for Social Issues Research, Study of Anxiety, Stress and Health Laboratory, University of Limerick, Limerick, Ireland; b Health Research Institute, University of Limerick, Castletroy, Limerick, Ireland; c Department of Psychology, University of Liverpool, Eleanor Rathbone Building, Bedford Street South, Liverpool, UK

The authors regret their names were incorrectly published. This has been corrected as above. The authors would like to apologise for any inconvenience caused.

